# High frequency opportunities for Alzheimer’s disease

**DOI:** 10.1093/braincomms/fcaf328

**Published:** 2025-09-08

**Authors:** Christos Panagiotis Lisgaras

**Affiliations:** Department of Psychiatry, New York University Grossman School of Medicine, New York, NY 10016, USA; Center for Dementia Research, the Nathan S. Kline Institute for Psychiatric Research, New York State Office of Mental Health, Orangeburg, NY 10962, USA

## Abstract

This scientific commentary refers to ‘High-frequency oscillations in epileptic and non-epileptic Alzheimer's disease patients and the differential effect of levetiracetam on the oscillations’, by Shandilya *et al*. (https://doi.org/10.1093/braincomms/fcaf041).

**This scientific commentary refers to ‘High-frequency oscillations in epileptic and non-epileptic Alzheimer's disease patients and the differential effect of levetiracetam on the oscillations’, by Shandilya *et al*. (https://doi.org/10.1093/braincomms/fcaf041).**

Alzheimer’s disease dementia has classically been defined by its hallmark pathological features, including extracellular amyloid-β (Aβ) plaques, intracellular neurofibrillary tangles comprised of hyperphosphorylated tau, and progressive neuronal loss leading to cognitive deficits. However, mounting evidence from both animal models and clinical studies suggests that these molecular alterations may only partially explain outcomes in Alzheimer’s disease and related dementias, including cognitive impairment. In this context, Alzheimer’s disease has increasingly been recognized as a disorder with prominent network dysfunction.^1^ Among these network alterations, hyperexcitability, a form of aberrant network activity typically characterized by increased neuronal firing, has been proposed as a significant contributor to disease progression and cognitive decline.^2^

Until recently, EEG indicators of network hyperexcitability in Alzheimer’s disease were largely limited to interictal epileptiform discharges (IEDs) and seizures. In epilepsy, a brain disorder frequently comorbid with Alzheimer’s disease, high frequency oscillations (HFOs) are common and considered a promising biomarker.^3^ However, it remained unclear whether HFOs occurred beyond the context of epilepsy or held relevance for Alzheimer’s disease. A study using mouse models of cerebral amyloid overexpression demonstrated that HFOs are present not only in the hippocampus but also on the cortical surface across the Alzheimer’s disease continuum, including both early and advanced stages of Alzheimer’s disease-related neuropathology.^4^ The recent study by Shandilya and colleagues^5^ in *Brain Communications* took a significant step forward by identifying HFOs as candidate biomarkers of hyperexcitability in humans with Alzheimer’s disease. Using high-resolution magnetoencephalography (MEG) in a randomized, double-blind clinical trial of the anti-seizure drug levetiracetam, the authors provided evidence that HFOs are detectable in Alzheimer’s disease and are differentially modulated by levetiracetam depending on the presence or absence of epileptiform activity.

## Wideband MEG identifies HFOs in all Alzheimer’s disease cases

**Figure 1:**
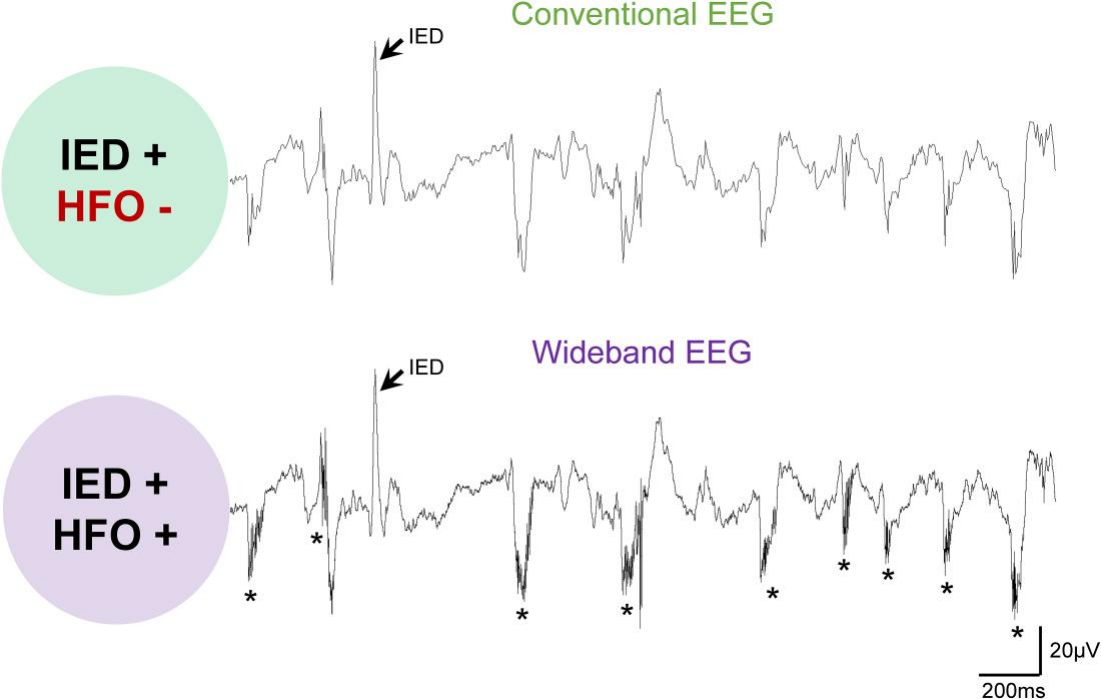
**Comparison of conventional versus wideband EEG in detecting HFOs and IEDs.** The upper trace shows a signal sampled at 500 Hz with a 1–70 Hz bandpass filter, representative of a conventional EEG setting. An IED (marked by a black arrow) is visible, but no HFO activity is detected (IED+, HFO−). The lower trace shows a signal recorded using wideband settings (2000 Hz sampling rate; 0.1–500 Hz bandpass filter). Several HFOs (marked by asterisks) are now evident, along with the same IED (IED+, HFO+). The signal comes from a mouse model of temporal lobe epilepsy (intrahippocampal kainic acid model) and is shown for illustrative purposes. The top trace was generated by downsampling and filtering the original wideband signal shown in the bottom trace.

The study design brought several innovations.^5^ They used a 275-channel whole-head system with high sampling rate (1200–4000 Hz), allowing for multi-channel detection of HFOs in a non-invasive manner. Three participant groups were examined: (1) subjects with Alzheimer’s disease without epileptic activity (non-epileptic Alzheimer’s disease), (2) subjects with Alzheimer’s disease and sub-clinical epileptic activity (epileptic Alzheimer’s disease), along with (3) healthy controls. Two detection algorithms, Delphos and a custom-developed pipeline, were used to ensure HFO detection validity. HFOs were analysed separately within the ripple (80–250 Hz) and fast ripple (250–500 Hz) frequency bands. During baseline recordings, all participants with Alzheimer’s disease as well as healthy controls showed ripples and fast ripples, but the Alzheimer’s disease cases showed more than healthy controls. That was the case across several regions, including frontal, temporal, and parietal areas. In contrast, only 57.1% of participants with Alzheimer’s disease had IEDs, suggesting that HFO analysis may identify a broader population with underlying network hyperexcitability. Similar results were observed in an independent cohort of individuals with Down syndrome, who are at ultra-high risk of developing Alzheimer’s disease dementia.^6^ Collectively, these findings highlight the utility of wideband recordings capable of capturing rapid electrophysiological events in identifying individuals who exhibit abnormal electrical activity, but at faster frequencies compared with the range of frequencies that are currently used clinically, the classic Berger frequencies (i.e. 1–70 Hz; Fig. 1). This is particularly important given prior concerns that hyperexcitability in Alzheimer’s disease may be underestimated using brief recordings^7^ or the inability to sample deep brain structures such as the hippocampus.^8^ Remarkably, the authors were able to detect HFOs in every single participant from just a 10 min MEG recording. This finding raises the intriguing possibility that HFOs may be more frequent than IEDs in Alzheimer’s disease. Although a direct comparison between HFO versus IED rates is still needed, evidence from mouse models suggests that HFOs consistently outnumber IEDs across vigilance states, including wakefulness and sleep.^9^ Still, more studies are necessary to determine the optimal recording duration needed to reliably detect HFOs in Alzheimer’s disease, given their known variability in both time and space, a challenge that has been recognized in epilepsy.^10^

## Divergent effects of levetiracetam in Alzheimer’s disease subtypes

An intriguing finding from this study^5^ was that the effect of levetiracetam on HFOs depended on the presence or absence of epileptiform activity. Specifically, levetiracetam increased both ripples and fast ripples in non-epileptic Alzheimer’s disease cases, whereas it was associated with decreased rates in cases with detectable epileptiform activity. What might account for these opposing effects? The authors discuss these divergent responses in relation to potential differences in SV2A expression, the synaptic vesicle protein targeted by levetiracetam, and/or variations in ion channel expression between Alzheimer’s disease subtypes. An alternative explanation could be that levetiracetam’s impact on HFOs may be mediated by its known effects on IEDs.^11^ For instance, if some individuals with epileptic Alzheimer’s disease show elevated HFO rates because some of these HFOs occur during IEDs, as has been observed in epilepsy^12^ and Down syndrome-associated Alzheimer’s disease,^4,6^ then a reduction in IEDs by levetiracetam^11^ could secondarily lower HFO counts. However, this mechanism would not explain the observed increase in HFO rates in participants with no robust evidence of IEDs, as determined by overnight EEG and 1 h MEG. A study in Alzheimer’s disease mouse models proposed a competing relationship between IEDs and HFOs, wherein elevated HFO rates might suppress the occurrence of IEDs.^9^ If that is the case, levetiracetam-induced suppression of IEDs could relieve this competition, allowing HFOs to become even more prominent. These observations highlight the complex nature of interpreting drug effects on network-level biomarkers and emphasize the need for future studies to also consider potential interactions among electrophysiological signatures in the context of both Alzheimer’s disease and epilepsy.

## Implications for the role of HFOs in epilepsy

HFOs have been an exciting theme of epilepsy research for the past two decades, culminating in their designation as one of the most promising biomarkers of epileptogenicity.^3^ However, their clinical utility has increasingly been debated, as HFOs do not consistently predict outcomes at the individual level.^13^ For instance, findings from a randomized controlled trial demonstrated that HFOs were not superior to IEDs in predicting post-surgical outcome, albeit they hold promise for extratemporal epilepsies.^14^ The recent detection of HFOs in neuropsychiatric conditions^5,6^ independently of a formal epilepsy diagnosis, could lead us to suggest that HFOs are not specific to epilepsy as in the past.^4^ However, rather than reflecting a lack of specificity, these findings also prompt a critical reevaluation of the role of hyperexcitability in neurodegenerative disease. One possibility is that these populations, such as individuals with Alzheimer’s disease or Down syndrome, are at ultra-high risk for epilepsy or may even harbor undiagnosed forms of the disorder. If HFOs in Alzheimer’s disease and Down syndrome indeed reflect underlying hyperexcitability, this would support the prediction that network dysfunction in these conditions extends beyond what is detectable by conventional (non-wideband) EEG. As such, future efforts should prioritize wideband electrophysiological approaches capable of capturing fast, transient, presumably pathological activity that traditional EEG systems, typically limited to the 1–70 Hz frequency range, are not designed to detect.
